# Balance assessment in selected stages of Parkinson’s disease using trend change analysis

**DOI:** 10.1186/s12984-023-01229-1

**Published:** 2023-08-02

**Authors:** Piotr Wodarski, Jacek Jurkojć, Justyna Michalska, Anna Kamieniarz, Grzegorz Juras, Marek Gzik

**Affiliations:** 1 Department of Biomechatronics, Faculty of Biomedical Engineering, Silesian University of Technology, Gliwice, Poland; 2 Department of Human Motor Behavior, Institute of Sport Sciences, Academy of Physical Education in Katowice, Katowice, Poland

**Keywords:** Parkinson disease, Trend Change Index, Body balance, Postural stability, Posturography

## Abstract

**Background:**

Balance disorders in patients diagnosed with Parkinson’s disease (PD) are associated with a change in balance-keeping strategy and reflex disorders which regulate the maintenance of vertical body posture. Center of foot pressure (COP) displacement signals were analyzed during quiet standing experiments to define such changes. The research aimed to apply stock exchange indices based on the trend change analyses to the assessment of a level of the Parkinson disease progression on the grounds of the analysis of the COP signals.

**Methods:**

30 patients in two stages of PD, 40 elderly participants, and 20 individuals at a young age were studied. Each person was subjected to 3 measurements with open and closed eyes. A technical analysis of the COP displacement signal was performed, and the following quantities were determined: indices related to the number of trend changes (TCI), indices defining a mean time (TCI_dT), and mean displacement (TCI_dS) and mean velocity (TCI_dV) between such changes.

**Results:**

The results indicate a higher TCI value for PD than for aged-matched control group (p < 0.05). In the case of PD patients, there was also an increase in the TCI_dS value by 2–5 mm, which mainly contributed to the increase in TCI_dV. Statistically significant differences for the TCI_dT values occurred between all groups in which differences in the average COP velocity were noted.

**Conclusions:**

The TCI and TCI_dV results obtained for the healthy participants enabled the development of indices supporting PD diagnostics. The causes of the TCI_dV changes in patients were determined, i.e., whether they resulted from an increase in the TCI_dT or TCI_dS between the moments of trend changes indicated by the developed algorithm. The developed methodology provides new information on the impact of PD on the strategy of maintaining balance, which was impossible to obtain using currently used analyses.

*Trial registration* The conducted research is an observational study and does not include a health care intervention. Participants gave their consent to participate in the research and the procedure was approved by the Institutional Bioethics Committee.

## Introduction

Alterations or disorders of postural control are related to involuntary changes taking place inside the human body. Aging processes are accompanied by progressive degeneration of all functional and anatomical systems [1, 2]. Balance disorders affect not only elderly people but also patients suffering from neurodegenerative illnesses, including Parkinson’s disease (PD) [3]. The effects of the disease may affect a patient’s mental or physical character and adopt various forms. The most common symptoms of PD include resting tremor, rigidity, bradykinesia as well as disorders of gait and balance. All these factors make patients with PD more susceptible to balance loss, falling and, subsequently, serious injuries [4, 5].

Standard diagnostics of balance in patients with PD are based on simple functional tests, including the Unified Parkinson’s Disease Rating Scale (UPDRS), Berg Balance Scale (BBS), Tinetti Balance and Gait Scale, retropulsion test, and the Functional Reach test, which provide the basis for the evaluation of balance disorders leading to an increase in the risk of falls [6–8]. These functional tests assess visible and fixed long-term disorders of the body posture. However, in the case of individuals suffering from neurodegenerative diseases, early and reliable diagnosis is of crucial importance, as it enables adequate intervention. The way a functional test in clinical practice is performed and evaluated often has a subjective character, which results in a qualitative assessment [4]. However, quantitative methods to evaluate balance disorders may be used. Moreover, the quantitative assessment gold standard of the body’s balance and stability is provided by posturography tests, which enable the analysis of the center of foot pressure (COP) displacement. On this basis, the following variables are determined: length of the COP path, ranges of the COP displacements, or ellipse surface area. Quiet standing (QS) is the most frequently used test for the evaluation of static balance and can be performed with or without the control of vision. The QS test is widely applied to the balance diagnostics of patients with neurodegenerative diseases, as described by Marchese et al. [9], Horak et al. [5], and Błaszczyk et al. [10]. Differences in the posturographic variables between patients with PD and the age-matched control group made it possible to discern balance disorders. However, the assessment of balance disorders based on standard posturographic variables does not fully enable early diagnosis because the postural quantities do not correlate with the duration or intensity of PD [11, 12]. This shortcoming prompted the modifications of QS tests to provide a more functional test, which could be performed on a force plate, such as investigations conducted by Termoz et al. [13]. In these tests, a modification of the feet position during the postural balance measurements was undertaken, which increased the differences between the measured mean values, such as the COP path length and the COP displacement for ill persons in relation to healthy individuals. Another example is the research by Oude Nijhuis et al. [14] and Johnson et al. [8], who introduced an additional measurement on force plates with the movement having several degrees of freedom, or a test of maximum sway in several directions resulting in the indication of patients more prone to falls. More often, the evaluation of early balance disorders is performed using a dual task. After introducing a second task during the QS test, which involved the enumeration of the greatest number of animal species or the largest number of words starting with the letter R, patients with PD revealed a greater swinging of posture in the medio-lateral (ML) plane than in the anterior-posterior (AP) plane [15]. Furthermore, such patients significantly differed from the control group.

Apart from balance disorders, one of the main symptoms of PD is gait disorder. That is the reason why quantitative methods of assessing balance disorders are connected not only with statics but also with dynamic conditions [16]. In the research by Shin and Ahn [17], the authors explicitly point out the asymmetry appearing between the lower limbs during gait. A decrease in the length of the COP trajectory in the stance phase was observed on the side which was affected by PD. Postural stability during the step and gait initiation seems to be an important predictor of dynamic balance disorders [18–20]. A prolonged time of reaction, reduced step length, or prolonged time of regaining a stable body posture after the performance of a step is typical of patients diagnosed with PD.

Balance disorders in patients with PD are related to the change in balance-maintaining strategy with disorders of reflexes regulating the vertical body posture [5]. The above-mentioned strategy consists of increased awareness of performed postural corrections during standing or walking [21]. A patient’s vision is beginning to play a more extensive role in postural control [22]. In addition, while standing, patients move their COP forward on the ground, which indicates an increased rigidity of ankle muscles [16, 21]. The increased rigidity of muscles and postural reflexes contributes to the impairment of balance control, thus influencing the frequency of postural corrections. The above-mentioned changes, indicated by Rinalduzzi et al. [21] and by Mirelman et al. [16], potentially induced researchers to investigate and analyze the COP signal within the scope of seeking repeated periodic patterns leading to the analyses in the frequency domain. Tests using fast Fourier transform (FFT) analysis and computer-aided models based on artificial intelligence-enabled the detection of PD at an early stage. The findings contributing to early PD detection are presented in the studies by Rezvanian et al. [23]—the selection of Motor Subtypes and Rana et al. [24] as well as Fadil et al. [25]—the discovery of repeated patterns in measured physiological signals and the COP signal leading to new ways of their detection.

Analyses in the frequency domain based on the detection of the change in the direction of the foot pressure displacement signal seem to be an adequate diagnostic tool for the detection of PD phases. The FFT method is difficult to interpret and poses some drawbacks, such as a spectral leak, whose elimination is connected with the prolongation of the measurement duration [22, 26]. The application of FFT makes it possible to detect periodic features of the signal and omit short-term trend changes; harmonic values of tiny changes are covered by spectral noise [22, 27]. The analyses of trend changes seem to be a slightly better method of detecting COP signal direction changes. The methodology proposed by the authors in their previous work enables the identification of vital trend changes in the COP movement, i.e., changes in the direction of the COP movement that are not of a short-term character, which are insignificant in terms of the conducted analyses [26, 28]. The base signal is processed in the time domain. In doing so, the calculated indices omit mistakes in the FFT computations (ex. spectral leakage). The results may be presented in histograms of trend changes, which are connected with the results obtained in the frequency analyses.

## Methods

### The aim of the study

The conducted investigations aim to utilize stock exchange indices based on trend change analyses in the evaluation of the PD stages using COP displacement signals during the activity of QS. The determination of dependences connected with the frequency of the occurrence of trend changes, their number, and the COP displacement between the changes appear to be a useful tool in the assessment of human balance in different phases of PD. The studies involved performing standing tests with open eyes (EO) and closed eyes (EC), which are standard in the evaluation of balance and constitute an essential part of the methodology for diagnostics preventing patients from falls [29, 30].

One can assume that postural disorders caused by PD lead to an increased number of postural corrections determined as trend changes in the COP movement as well as increases in the mean value of the COP velocity between subsequent trend changes. We believe it is possible to indicate whether an increase in the COP velocity results from a distances of momentary sways of the posture, which may, in turn, indicate increased balance disorders in PD patients. In the case of positive verification of the formulated theses, the application of stock exchange indices to stabilographic tests may enable further development of methods diagnosing balance disorders in individuals with PD by showing trend changes in these disorders associated with the progression of this disease.

### Study group and experimental procedure

The experimental groups consisted of 30 subjects with idiopathic PD, 40 age-matched, healthy control subjects, and 20 young, healthy control subjects. The demographic and clinical characteristics of the subjects are presented in Table 1.

**Table 1:** Characteristics of test groups (*indicates a statistically significant differences between CGy and CG, CGy and PDII, CGy and PDIII, ^+^indicates a statistically significant differences between CGy and PDII, CGy and PDIII)

Designation	CGy	CG	PDII	PDIII
Designation description	Healthy control subjects	Aged-matched healthy control subjects	A group of people with stage II Parkinson’s disease	A group of people with stage III Parkinson’s disease
Group size	20 (8 male, 12 female)	40 (8 male, 32 female)	15 (11 male, 3 female)	15 (11 male, 3 female)
Age (years)	20 ± 1.1*	69 ± 5.5	63 ± 7.2	69 ± 8.6
Body mass (kg)	63.9 ± 11^+^	71.6 ± 13	82 ± 11	83 ± 20
Body height (cm)	171 ± 7.2	163 ± 8.4	172 ± 8	170 ± 8

Based on a medical interview, the general inclusion criteria for PD subjects and aged-matched, healthy control subjects (CG) were: age ≥ 55 years, including PD subjects identified and diagnosed with PD stage II or III according to the Hoehn and Yahr scale, UK Brain bank criteria. For young, healthy control subjects (CGy), not practicing sports professionally, the inclusion criteria were: age between 19 and 25. The exclusion criteria for all research subjects were as follows: neuromuscular, vestibular, or orthopedic disorders. Additionally, for PD and CG subjects the exclusion criteria were: dementia or cognitive impairment (MMSE score below 26 points). PD subjects were tested during the “ON period” of their usual anti-parkinsonian medication (at least 1 h). The study was approved by the Institutional Bioethics Committee AWF Katowice (7/2013).

**Fig. 1:**
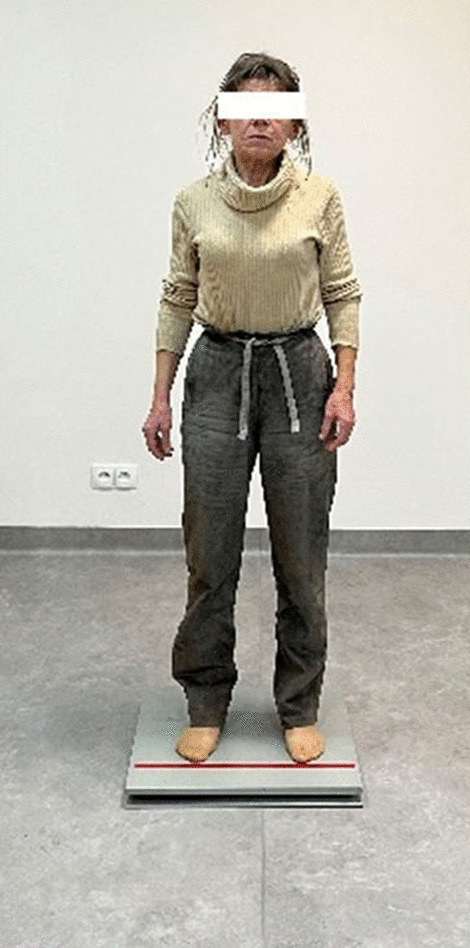
Measurement stand with a measurement platform

Subjects performed two balance tasks while standing barefoot on a force plate with EO and EC. They were asked to stand in a comfortable position with their feet approximately shoulder-width apart on the force platform, with their gaze fixated at a reference point located 5 m away in front of them (QS). Each trial lasted 30 s and was repeated three times. The order of the trials was randomized for each subject (Fig. 1).

The data was recorded using a force plate (AMTI, AccuGait). The platform sampling frequency was 100 Hz. A dual-pass 7 Hz low-pass Butterworth filter was used for forces (Fx, Fy, Fz) and moments (Mx, My, Mz), which were later used to calculate the COP. All calculations were performed using MATLAB software (Mathworks, Natick, MA).

### Analysis of the results

The information obtained from the stabilographic platform included the COP displacements in the AP and ML directions. Using the displacement information, the resultant velocity of the COP displacement (COP velocity, mm/s) was determined. In addition, the technical analysis of the COP signal was performed. In the first step, the EMA_9_, EMA_12_ and EMA_26_ signals were determined using dependence 1.

1 $${\text{EMA}}_{\text{pN}}=\frac{{\text{p}}_{0}+\left(1-{\upalpha }\right){\text{p}}_{1}+{(1-{\upalpha })}^{2}{\text{p}}_{2}+{(1-{\upalpha })}^{3}{\text{p}}_{3}+\cdots +{(1-\text{a})}^{\text{N}}{\text{p}}_{\text{N}}}{1+\left(1-{\upalpha }\right)+{(1-{\upalpha })}^{2}+{(1-{\upalpha })}^{3}+\cdots +{(1-{\upalpha })}^{\text{N}}}$$

where p0 = ultimate value; p1 = penultimate value; pN = value preceding N periods; N = number of periods.

The vector MACD_12,26_ was then determined as the difference between EMA_12_ and EMA_26_.

Finally, the MACD vector was determined as a vector of intersection points of MACD_12,26_ and EMA_9_ signals. The algorithm was described in detail in the authors’ previous work [26, 28]. The moments of the trend changes for the COP displacement signals were determined separately in the AP and ML directions.

The next phase involved the determination of the following values:

- MACD_dT—vector of time between subsequent trend changes (The vector contains the time differences between successive occurrences of trend changes on the COP signal).
- MACD_dS—vector of the COP displacement between subsequent trend changes (The vector contains the distance of the COP movement between successive changes in the trend of the COP signal).
- MACD_dV—vector of the COP movement velocity between subsequent trend changes calculated as a ratio of counterpart values in vector MACD_dS to vector MACD_dT.
- TCI—trend change index, total number of trend changes during the whole test.
- TCI_dT—mean value of determined vector MACD_dT (s).
- TCI_dS—mean value of determined vector MACD_dS (mm).
- TCI_dV—mean value of determined vector MACD_dV (mm/s).

**Fig. 2:**
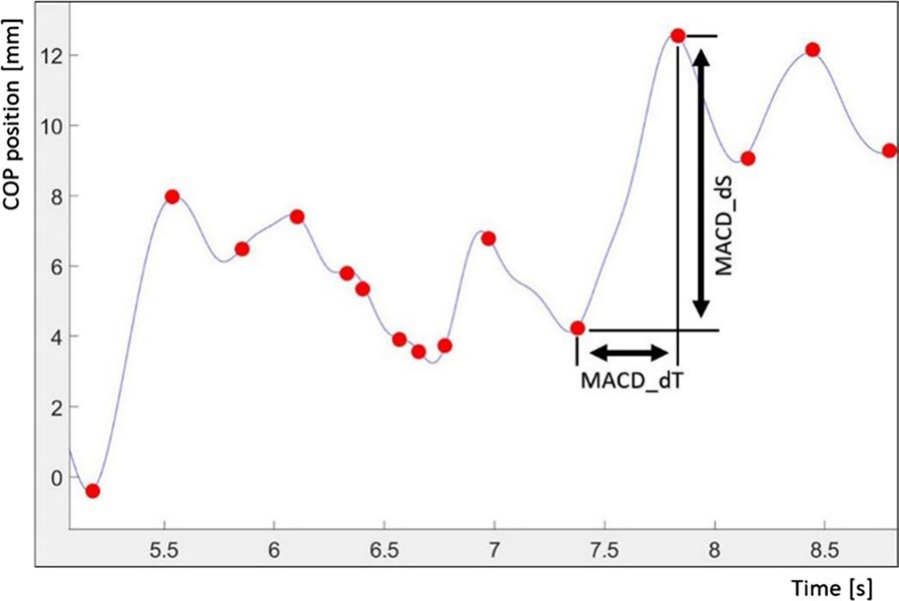
COP course with trend change moments detected by the algorithm (marked as red dots in the diagram) and graphic interpretation of MACD_dT and MACD_dS for two random trend change moments (100 Hz signal frequency initially filtered with a 7 Hz low-pass filter 7 Hz)

The graphic interpretation of MACD_dT and MACD_dS with the determined moments of trend changes is presented in Fig. 2.

All values TCI, TCI_dV, TCI_dT and TCI_dS were determined separately for the AP and ML directions. The next stage involved the determination of the TCI resultant values by summing up the results obtained for both directions. In the case of other values (TCI_dV, TCI_dT and TCI_dS), the sum of vector values for individual directions was adopted as the resultant value.

### Statistical analysis

All analyses were performed using Matlab R2022a and Statistica 13. To test normal distributions, the Shapiro-Wilk test was used. The homogeneity of variance was tested using the Brown-Forsythe test. The result obtained for the TCI showed the occurrence of normal distribution and homogeneity of variance in all subgroups. The statistical analysis for the TCI was performed using the ANOVA test, taking into consideration independent groups. The Scheffé test was applied as the post-hoc test. The strength of tests always amounted to more than 0.9.

In the case of the remaining analyzed values, no normal distribution or variance homogeneity was found in the tested groups. Based on this, the Kruskal–Wallis analysis of variance (ANOVA) was used, followed by Dunn’s post-hoc test. The reliability of the result was investigated using Dunn’s test following the methodology proposed by Rea and Parker [31], identifying r (epsilon square). The strength of tests always amounted to more than 0.74.

Intraclass correlation coefficients (ICC 2,1) were calculated for new posturographic parameter. The levels of reliability were considered poor (ICC < 0.40), moderate (0.40 ≤ ICC < 0.60), good (0.60 ≤ ICC < 0.80), and excellent (ICC ≥ 0.80) according to Mancini et al. [32].

## Results

For all of the determined values, reliability was calculated by taking into consideration three measurements for each person. The obtained interclass correlation coefficient (ICC 2,1) values in the case of the TCI were within the range of 0.80–0.92, whereas other values were in the range of 0.74–0.88. These ranges indicate the high reliability and repeatability of the conducted analyses.

**Fig. 3:**
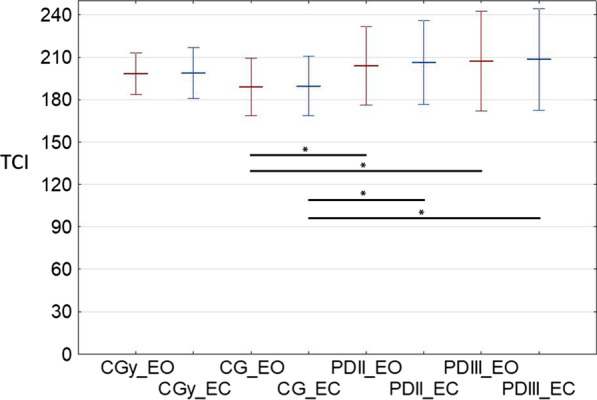
Calculated TCI values. Results show mean values increased and decreased by standard deviation. The asterisk (*) is used to represent a comparative statistically significant result. Tests with eyes open (EO) are marked in red and tests with eyes closed (EC) are marked in blue

Figure 3 presents the resultant value of the calculated TCI. Normal distributions were obtained for the calculated values. Therefore, the results are presented as mean values (± standard deviation).

In the conducted investigations, it were obtained a higher values of the proposed TCI in the cases of PD stages II and III than in the case of CG (Fig. 3; Table 2). The results were confirmed in the EC and EO conditions. In the case of patients with stage II and III PD, the number of postural corrections during the test did not statistically differ from the values obtained for CGy (Table 2).

**Fig. 4:**
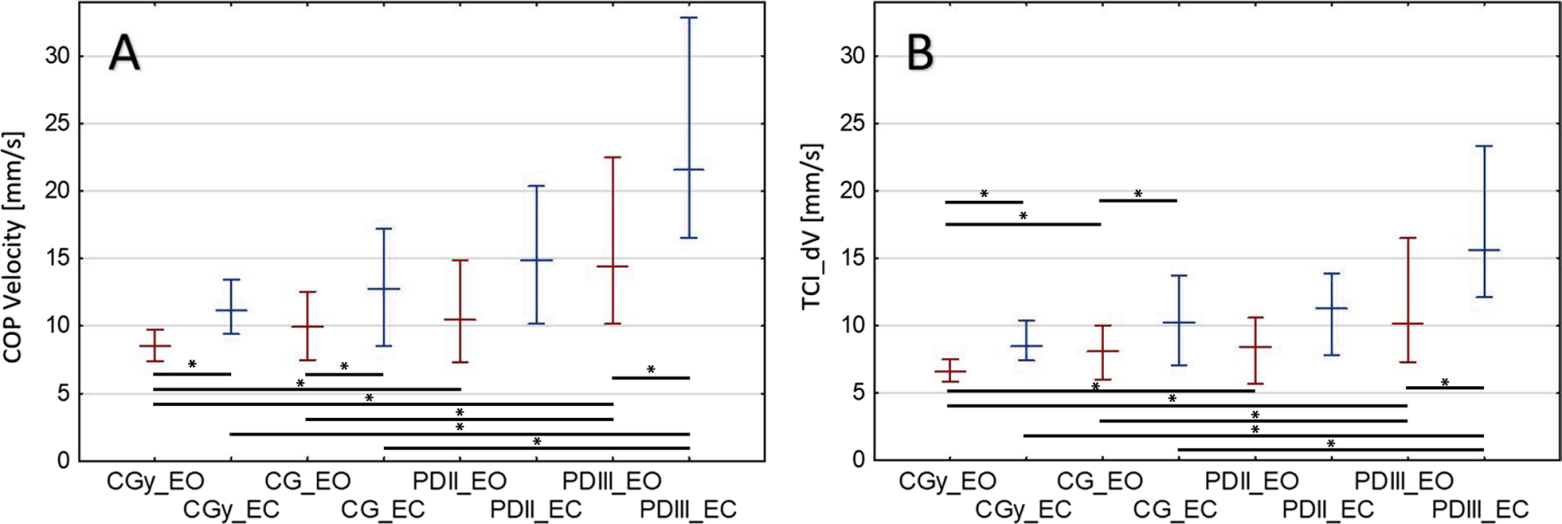
Calculated medians and interquartile distribution of the COP movement velocity (**A**) and TCI_dV (**B**). The asterisk (*) is used to represent a comparative statistically significant result. Tests with eyes open (EO) are marked in red and tests with eyes closed (EC) are marked in blue

Figure 4 presents the calculated resultant values of the COP displacement velocity during the QS test (Fig. 4A), and the TCI_dV values determined based on the technical analysis (Fig. 4B). The values did not reveal normal distributions in all groups, therefore the median and interquartile distribution were presented.

In Fig. 4A, one may observe an increase in the interquartile distribution in the case of stage III PD patients (at least twice as wide of a range) in relation to CG for both EO and EC. Moreover, there was an increase in the mean COP velocity in the case of stages II and III PD patients compared to CGy as well as in the case of stage III patients in relation to CG in the EO test. As for the EC test, the above-mentioned increase occurred only in the case of stage III PD patients compared to CGy and CG. Observations similar to the analysis of the COP velocity were observed in the case of the TCI_dV calculated using the trend change analysis (Fig. 4B). In terms of values, this index reached lower values in relation to the COP velocity. Statistically significant differences for TCI_dT values occurred between all groups where differences in the mean COP velocity were found while comparing tested groups, with one exception. In the case of the TCI_dV, statistically significant differences were also found between the CG and CGy groups tested under the EO conditions (Table 2).

**Fig. 5:**
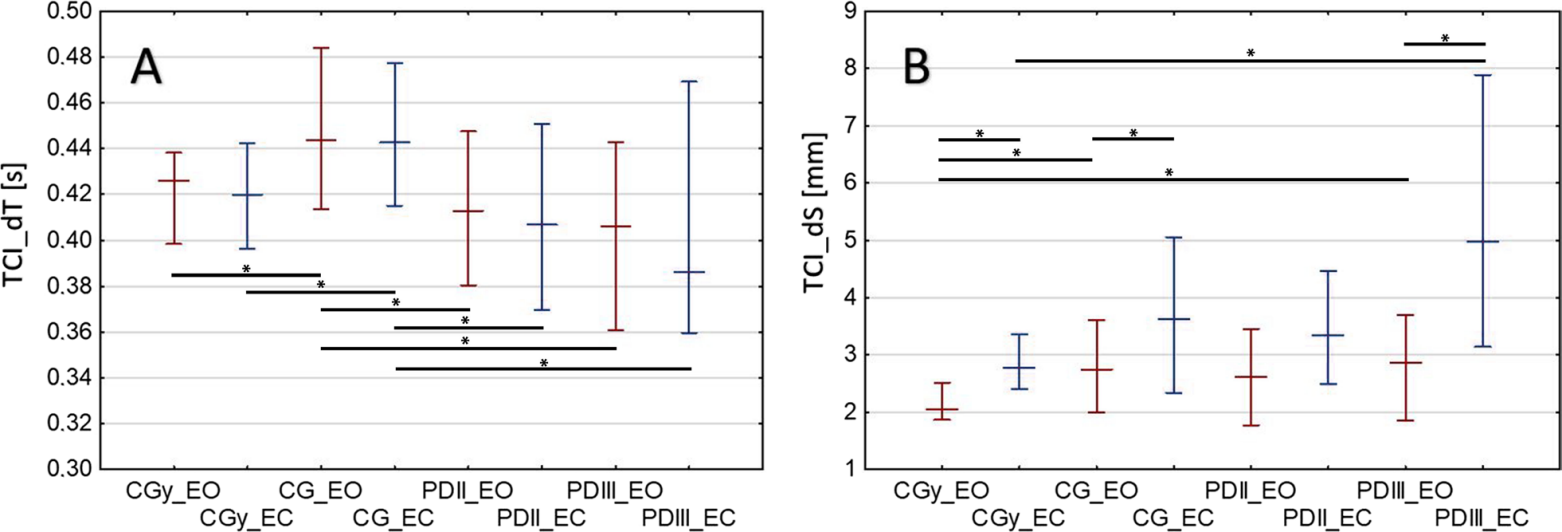
Calculated medians and interquartile distribution of TCI_dT (**A**) and TCI_dS (**B**). The asterisk (*) is used to represent a comparative statistically significant result. Tests with eyes open (EO) are marked in red and tests with eyes closed (EC) are marked in blue

Figure 5 presents the TCI_dT values (Fig. 5A) and the TCI_dS values (Fig. 5B), which were calculated based on the technical analysis. The above-mentioned values did not reveal normal distribution in all groups, hence the median and interquartile distribution were presented.

The median TCI_dT for stage II and stage III PD does not statistically differ from the CGy (Fig. 5A) and is less than the CG (Table 2). In the case of patients with stage III PD, there was an increase in the TCI_dS value (Fig. 5B), 5 mm and 2 mm for patients with stage III PD and CGy, respectively, which mainly influenced the increase of TCI_dV in the case of stage III PD.

The values presented in the diagrams were subjected to statistical analysis. The calculated p values obtained during the statistical computations are presented in Table 2. Statistically significant differences between groups have been marked with an asterisk.

**Table 2:** Results of the statistical analysis

Group and condition		P value				
		TCI	V COP	TCI_dV	TCI_dT	TCI_dS
CGy_EO	CG_ EO	0.554	0.218	0.022*	0.039*	0.001*
CGy_ EO	PDII_ EO	0.987	0.044*	0.048*	1.000	0.325
CGy_ EO	PDIII_ EO	0.825	0.000*	0.000*	0.943	0.020*
CG_ EO	PDII_ EO	0.049*	0.916	1.000	0.016*	0.954
CG_ EO	PDIII_ EO	0.010*	0.000*	0.008*	0.001*	1.000
PDII_ EO	PDIII_ EO	1.000	0.153	0.097	0.999	0.973
CGy_ EC	CG_ EC	0.601	0.826	0.531	0.035*	0.125
CGy_ EC	PDII_ EC	0.926	0.241	0.481	0.997	0.717
CGy_ EC	PDIII_ EC	0.763	0.020*	0.000*	0.921	0.001*
CG_ EC	PDII_ EC	0.035*	0.865	1.000	0.007*	0.999
CG_ EC	PDIII_ EC	0.007*	0.002*	0.001*	0.001*	0.279
PDII_ EC	PDIII_ EC	1.000	0.365	0.052	1.000	0.241
CGy_ EO	CGy_ EC	1.000	0.003*	0.001*	1.000	0.001*
CG_ EO	CG_ EC	1.000	0.001*	0.000*	1.000	0.001*
PDII_ EO	PDII_ EC	1.000	0.128	0.080	1.000	0.067
PDIII_ EO	PDIII_ EC	1.000	0.043*	0.042*	1.000	0.000*

Mean values and medians with statistically significant differences are marked with an asterisk

## Discussion

The application of a force platform measuring the ground reaction forces, based on which it is possible to determine the periodical positions of the COP, appears to be a useful tool for the evaluation of the postural balance of patients with PD [33, 34]. Changes in postural swinging may be described with ‘classic’ values, which are determined during the COP course. A more advanced analysis of the posturographic signal using the analyses in the frequency domain finds its application especially in the measurements making use of a cyclic factor upsetting the postural balance.

The application of trend change analyses to the COP movement based on algorithms used in the analysis of stock exchange rates constitutes an entirely new approach to the assessment of the ability to maintain balance. The results of computations make it possible to determine and evaluate not only the number of momentary corrections of posture but also indicate the changes affecting the distance covered by the COP during each correction of the posture and the duration of such corrections.

### Analysis of the TCI

Balance disorders are one of the most frequent symptoms of motor disorders in PD. Similarly, the progression of the disease affects the characteristics of postural sway [35, 36]. In accordance with the adopted Hoehn and Yahr classification, problems maintaining balance are characteristic of the third stage of the disease. However, more and more studies indicate that postural sway is subject to changes already taking place in the early phases of the disease, before the occurrence of clinical symptoms. The above-mentioned findings justify the search for a diagnostic method based on COP displacements [37]. One of the fundamental parameters used in balance tests is the mean velocity of the COP displacement. The value of this variable often differentiates patients with PD from the CG [33]. The conducted investigations (Fig. 4A; Table 2) confirm these results only when healthy individuals (both age groups) and stage III PD patients are compared, where an increase in the mean COP velocity was observed in the case of PD in both the EO and EC tests. As for the stage II PD patients, statistically significant differences were observed only in comparison with the CGy group in the EO test. This highlights that the differentiation between PD patients and the CG group occurs only in patients with later stages of PD (stage III). In the studies by Tadayoshi et al. [34] and Ferrazzoli et al. [38], the root mean square parameter determined for the mean COP velocity was indicated as the most significant factor, which directly translates into the standard deviation calculated for these values. Figure 4A also demonstrates that in the case of the calculated velocity, there is an increase in the interquartile distribution value for stage III PD patients compared to CG, both for the EO and EC tests. These results are also confirmed by the research done by Barbosa et al. [39] and Ickenstein et al. [40], who noticed the increase in the interquartile distribution of the COP velocity without taking into consideration the stages of the disease.

The TCI is a parameter that enables the extension of the conducted analyses and, at the same time, the improvement of the interpretation of the results using posturographic examinations. The computational results obtained based on the conducted tests indicate a statistically higher value of the proposed TCI for PD stages II and III than for CG (Table 2). The results were confirmed in the EC and EO tests. In the case of stages II and III of PD, the number of postural corrections during the test does not statistically differ from the value obtained in the case of CGy (Fig. 3). The determination of the TCI enabled better differentiation of individuals with PD in stages II and III in relation to CG and CGy.

### Analysis of momentary velocity, displacements and time between subsequent trend changes of the COP signal

The deterioration of balance-keeping ability in patients with PD may be observed in the changes of parameters, such as the mean COP velocity or an increase in the range of the COP movements [41, 42]. The analysis of these types of parameters indicates only global changes in relation to the total time of the measurement. Analyzing the TCI, TCI_dV, TCI_dT, and TCI_dS enables one to determine changes connected with momentary ‘leaps’ of the COP, defined as trend changes in the COP movement.

The analysis of the median values of the TCI_dV determined for subsequent groups of tested individuals indicates that they are subject to trend changes similar to the mean COP velocity. However, in terms of numerical values, TCI_dV reaches lower values than the COP velocity, which may result from the fact that the algorithm specifying the TCI filters out noises from the signal, i.e., omittable tiny changes in the COP position (Fig. 4B). Statistically significant differences occurred everywhere where there were differences in the mean COP velocity in the comparison of the tested groups, with one exception. The TCI_dV also exhibited differences between the CG and CGy test groups in the EO conditions. Taking this into consideration, the TCI_dV provides similar information to the analysis of the mean COP velocity for the entire examination.

The application of the TCI, TCI_dT, and TCI_dS indices constitutes a completely novel approach enabling the observation of changes taking place in the strategy of maintaining balance. The comparison of the CGy group with the CG group clearly demonstrates that in the CG group, there was a statistically significant prolongation of time (TCI_dT) in the EO and EC tests as well as the distance (TCI_dS) in the EO tests. This prolongation of time, i.e., the decrease in subsequent postural corrections, reflects the changing balance-keeping strategy and may indicate the declining role of the proprioceptive system in maintaining balance in favor of the organ of sight [43, 44].

In the case of patients with PD, an inverse trend in the TCI_dT changes is observed compared to the CG. The time periods between subsequent trend changes reach values that are observed in young people. In addition, patients with stage III PD revealed a much greater prolongation of TCI_dS and, in comparison with the CG, an increase in the TCI values. The observed changes in the TCI demonstrate that patients with PD reveal not only much more frequent postural corrections but also much shorter time periods of leaps between the subsequent COP positions, identified as trend changes. Statistically significant differences between the CG and patients with PD (stages II and III) make it possible to differentiate between healthy and ill individuals, which was previously not possible in the case of stage II PD patients only based on the mean COP velocity. In addition, the TCI_dS, which was calculated for the EC test, distinguishes between stage II and stage III PD groups in an unambiguous way, indicating that the disease progression is related to the extension of distance between the subsequent COP positions, identified as trend changes. Such an extension of the TCI_dS distance entailing an increase in the velocity of leaps of the COP to subsequent positions (TCI_dV) may also be an indication of a greater probability of the fall occurrence. Moreover, an unpredictable destabilizing factor combined with an increased value of the COP velocity may lead to such an increase in the velocity vector value that the deceleration of the body movement becomes impossible and results in the crossing of the stability boundary [42, 43]. All analysis we conducted with an assumption of lack of significant differences between kinematic values in women and men with PD [45, 46]. However, in the future, the presented research should be extended to the differentiation between women and men, as there are also reports indicating the possibility of difference in these two groups [47].

## Conclusions

The conducted investigations provide new valuable information within the scope of balance analysis after extracting particular features of the COP displacement signal. Thanks to the analysis of trend changes, it is possible to determine the number of postural corrections which result in a change in the direction of the COP displacement. The tests provide information about the changes in the number of postural corrections, which are reflected in the trend changes of the COP signal in the case of patients with stage II and III PD. The comparison of the obtained results with healthy individuals makes it possible to classify the developed TCI as a method supporting the diagnosis of PD.

The developed methodology also enabled the detection of changes in the COP displacement velocity in the tested groups. It is possible to identify the causes of velocity changes, whether such changes result from an increase in the mean time, or potentially from the increase in displacement between moments of trend changes indicated by the developed algorithm.

Despite obtaining a lot of new information using the developed methodology, it was not possible to unambiguously indicate differences between patients in different stages of PD. Further research should be conducted with particular attention paid to the differences in the obtained parameter values between the EO and EC tests. A small number of patients in the tested groups made it impossible to confirm, for all tested groups, the impact of the closure of eyes on the increase in the COP displacement velocity resulting from an increase in the displacement between the moments of trend changes. Future research should be supplemented with traditional clinical tests such as the Berg Balance Scale, Functional Reach, and Time Up and Go tests, dedicated to older subjects and patients with PD, to complement the motor characteristics of the group.

## Data Availability

Calculation results are available on the website: http://www.biomechanik.pl/extraMaterials/MatPD.zip.

## Abbreviations

PD: Parkinson disease
CGy: Healthy young control subjects
CG: Aged-matched healthy control subjects
PDII: A group of people with stage II Parkinson’s disease
PDIII: A group of people with stage III Parkinson’s disease
AP: Anterior–posterior
ML: Medio-lateral
FFT: Fast Fourier transform
COP: Center of foot pressure
QS: Quiet standing
EO: Opened eyes
EC: Closed eyes
ICC: Interclass correlation coefficient
TCI: Total number of trend changes during the whole test
TCI_dT: Mean value of determined vector MACD_dT
TCI_dS: mean value of determined vector MACD_dS
TCI_dV: Mean value of determined vector MACD_dV
MACD_dT: Vector of time between subsequent trend changes
MACD_dS: Vector of the COP displacement between subsequent trend changes
MACD_dV: Vector of the COP movement velocity between subsequent trend changes calculated as a ratio of counterpart values in vector MACD_dS to vector MACD_dT

## References

[1] Błaszczyk JW, Czerwosz L (2005). Postural stability in the process of aging. Gerontol Pol.

[2] Soto-Varela A, Rossi-Izquierdo M, Faraldo-García A, Vaamonde-Sánchez-Andrade I, Gayoso-Diz P, Del-Río-Valeiras M, Lirola-Delgado A, Santos-Pérez S (2016). Balance disorders in the elderly: does instability increase over time?. Ann Otol Rhinol Laryngol.

[3] Schoneburg B, Mancini M, Horak F, Nutt JG (2013). Framework for understanding balance dysfunction in Parkinson’s disease. Mov Disord.

[4] Souza CO, Voos MC, Barbosa AF, Chen J, Francato DCV, Milosevic M, Popovic M, Fonoff ET, Chien HF, Barbosa ER (2019). Relationship between posturography, clinical balance and executive function in Parkinson’s disease. J Mot Behav.

[5] Horak FB, Dimitrova D, Nutt JG (2005). Direction-specific postural instability in subjects with Parkinson’s disease. Exp Neurol.

[6] Józefowicz-Korczyńska M, Chmielecka-Rutkowska J, Mazerant A (2016). Clinical tests for balance stability and gait assessment—bedside tests. Pol Prz Otorynolaryngol.

[7] Opara J, Małecki A, Małecka E, Socha T (2017). Motor assessment in Parkinson’s disease. Ann Agric Environ Med.

[8] Johnson L, James I, Rodrigues J, Stell R, Thickbroom G, Mastaglia F (2013). Clinical and posturographic correlates of falling in Parkinson’s disease. Mov Disord.

[9] Marchese R, Bove M, Abbruzzese G (2003). Effect of cognitive and motor tasks on postural stability in Parkinson’s disease: a posturographic study. Mov Disord.

[10] Błaszczyk JW, Orawiec R, Duda-Kłodowska D, Opala G (2007). Assessment of postural instability in patients with Parkinson’s disease. Exp Brain Res.

[11] Chastan N, Debono B, Maltête D, Weber J (2008). Discordance between measured postural instability and absence of clinical symptoms in Parkinson’s disease patients in the early stages of the disease. Mov Disord.

[12] Fernandes Â, Coelho T, Vitória A, Ferreira A, Santos R, Rocha N, Fernandes L, Tavares JM (2015). Standing balance in individuals with Parkinson’s disease during single and dual-task conditions. Gait Posture.

[13] Termoz N, Halliday SE, Winter DA, Frank JS, Patla AE, Prince F (2008). The control of upright stance in young, elderly and persons with Parkinson’s disease. Gait Posture.

[14] Oude Nijhuis LB, Allum JH, Nanhoe-Mahabier W, Bloem BR (2014). Influence of perturbation velocity on balance control in Parkinson’s disease. PLoS ONE.

[15] Fernandes Â, Coelho T, Vitória A (2015). Standing balance in individuals with Parkinson’s disease during single and dual-task conditions. Gait Posture.

[16] Mirelman A, Bonato P, Camicioli R, Ellis TD, Giladi N, Hamilton JL, Hass CJ, Hausdorff JM, Pelosin E, Almeida QJ (2019). Gait impairments in Parkinson’s disease. Lancet Neurol.

[17] Shin C, Ahn TB (2020). Asymmetric dynamic center-of-pressure in Parkinson’s disease. J Neurol Sci.

[18] Juras G, Kamieniarz A, Michalska J, Słomka K (2020). Assessment of dynamic balance during step initiation in Parkinson’s disease patients and elderly—a validity study. Acta Bioeng Biomech.

[19] Kamieniarz A, Michalska J, Marszałek W, Akbaş A, Słomka KJ, Krzak-Kubica A, Rudzińska-Bar M, Juras G (2020). Transitional locomotor tasks in people with mild to moderate Parkinson’s disease. Front Neurol.

[20] Palmisano C, Beccaria L, Haufe S, Volkmann J, Pezzoli G, Isaias IU (2022). Gait initiation impairment in patients with Parkinson’s disease and freezing of Gait. Bioengineering (Basel).

[21] Rinalduzzi S, Trompetto C, Marinelli L, Alibardi A, Missori P, Fattapposta F, Pierelli F, Currà A (2015). Balance dysfunction in Parkinson’s disease. Biomed Res Int.

[22] Wodarski P, Jurkojć J, Gzik M (2020). Wavelet decomposition in analysis of impact of virtual reality head mounted display systems on postural stability. Sensors (Basel).

[23] Rezvanian S, Lockhart T, Frames C, Soangra R, Lieberman A (2018). Motor subtypes of Parkinson’s disease can be identified by frequency component of postural stability. Sensors (Basel).

[24] Rana A, Dumka A, Singh R, Panda MK, Priyadarshi N (2022). A computerized analysis with machine learning techniques for the diagnosis of Parkinson’s disease: past studies and future perspectives. Diagnostics (Basel).

[25] Fadil R, Huether A, Brunnemer R, Blaber AP, Lou JS, Tavakolian K. Early detection of Parkinson’s disease using center of pressure data and machine learning. In: 2021 43rd annual international conference of the IEEE engineering in medicine & biology society (EMBC). IEEE; 2021. p. 2433–6. 10.1109/EMBC46164.2021.9630451 34891772

[26] Wodarski P, Chmura M, Gruszka G, Romanek J, Jurkojć J (2022). The stock market indexes in research on human balance. Acta Bioeng Biomech.

[27] Sozzi S, Nardone A, Schieppati M (2021). Specific posture-stabilising effects of vision and touch are revealed by distinct changes of body oscillation frequencies. Front Neurol.

[28] Wodarski P, Chmura M, Gzik M, Gruszka G, Jurkojć J. Technical analysis of the displacements of the centre of pressure in the standing posture based on data obtained using selected stock market indicators. In: 2021 international conference BIOMDLORE; 2021. p. 1–6.

[29] Quijoux F, Nicolaï A, Chairi I, Bargiotas I, Ricard D, Yelnik A, Oudre L, Bertin-Hugault F, Vidal PP, Vayatis N, Buffat S, Audiffren J (2021). A review of center of pressure (COP) variables to quantify standing balance in elderly people: algorithms and open-access code. Physiol Rep.

[30] Mańko G, Kocot I, Pieniążek M, Sosulska A, Piwowar H (2016). Assessment the risk of falls versus postural stability of the elderly, using a S tabilographic platform. Secur Dimens Int Natl Stud.

[31] Rea LM, Parker RA (1992). Designing and conducting survey research: a comprehensive guide.

[32] Mancini M, Salarian A, Carlson-Kuhta P, Zampieri C, King L, Chiari L, Horak FB (2012). ISway: a sensitive, valid and reliable measure of postural control. J Neuroeng Rehabil.

[33] Rocchi L, Chiari L, Cappello A, Horak FB (2006). Identification of distinct characteristics of postural sway in Parkinson’s disease: a feature selection procedure based on principal component analysis. Neurosci Lett.

[34] Minamisawa T, Takakura K, Yamaguchi T (2009). Detrended fluctuation analysis of temporal variation of the center of pressure (COP) during quiet standing in Parkinsonian patients. J Phys Therapy Sci.

[35] Kamieniarz A, Michalska J, Brachman A, Pawłowski M, Słomka KJ, Juras G (2018). A posturographic procedure assessing balance disorders in Parkinson’s disease: a systematic review. Clin Interv Aging.

[36] Feigin VL, Krishnamurthi RV, Theadom AM, Abajobir AA, Mishra SR, Ahmed MB (2017). Global, regional, and national burden of neurological disorders during 1990–2015: a systematic analysis for the global burden of disease study 2015. Lancet Neurol.

[37] Beuter A, Hernández R, Rigal R, Modolo J, Blanchet PJ (2008). Postural sway and effect of levodopa in early Parkinson’s disease. Can J Neurol Sci.

[38] Ferrazzoli D, Fasano A, Maestri R, Bera R, Palamara G, Ghilardi MF, Pezzoli G, Frazzitta G (2015). Balance dysfunction in Parkinson’s disease: the role of posturography in developing a rehabilitation program. Parkinsons Dis.

[39] Barbosa AF, Souza Cde O, Chen J, Francato DV, Caromano FA, Chien HF, Barbosa ER, Greve JM, Voos MC (2015). The competition with a concurrent cognitive task affects posturographic measures in patients with Parkinson disease. Arq Neuropsiquiatr.

[40] Ickenstein GW, Ambach H, Klöditz A, Koch H, Isenmann S, Reichmann H, Ziemssen T (2012). Static posturography in aging and Parkinson’s disease. Front Aging Neurosci.

[41] Masani K, Vette AH, Abe MO, Nakazawa K (2014). Center of pressure velocity reflects body acceleration rather than body velocity during quiet standing. Gait Posture.

[42] Roman-Liu D (2018). Age-related changes in the range and velocity of postural sway. Arch Gerontol Geriatr.

[43] Student J, Engel D, Timmermann L, Bremmer F, Waldthaler J (2022). Visual perturbation suggests increased effort to maintain balance in early stages of Parkinson’s to be an effect of age rather than disease. Front Hum Neurosci.

[44] Godinho C, Domingos J, Cunha G, Santos AT, Fernandes RM, Abreu D (2015). A systematic review of the characteristics and validity of monitoring technologies to assess Parkinson’s disease. J Neuroeng Rehabil.

[45] Georgiev D, Hamberg K, Hariz M, Forsgren L, Hariz GM (2017). Gender differences in Parkinson’s disease: a clinical perspective. Acta Neurol Scand.

[46] Kang KW, Choi SM, Kim BC (2022). Gender differences in motor and non-motor symptoms in early Parkinson disease. Medicine (Baltim).

[47] Szewczyk-Krolikowski K, Tomlinson P, Nithi K (2014). The influence of age and gender on motor and non-motor features of early Parkinson’s disease: initial findings from the Oxford Parkinson Disease Center (OPDC) discovery cohort. Parkinsonism Relat Disord.

